# Personality Disorders and Clinical Response to Intranasal Esketamine in Treatment-Resistant Depression: Trajectories, Modulations, and Psychopathological Reflections

**DOI:** 10.1192/j.eurpsy.2026.10617

**Published:** 2026-07-31

**Authors:** V. Casati, K. La Monica, M. C. Angeletti, G. Versaci, A. Frediani, T. Prodi, F. Mazzoni, G. Miacca, A. Guffanti, N. Brondino, V. Martiadis, R. Anniverno, B. Dell’osso, M. Olivola

**Affiliations:** 1 Department of Mental Health and Addiction Services, ASST Fatebenefratelli Sacco, Milan; 2 Department of Mental Health, Department of Biomedical and Clinical Sciences Luigi Sacco, University of Milan; 3 Department of Mental Health and Addiction Services, ASST Fatebenefratelli Sacco, Milan; 4 Department of Mental Health, Department of Biomedical and Clinical Sciences Luigi Sacco, University of Milan, Milan; 5 Mental Health Department, ASST Pavia, Pavia; 6 Mental Health Department, ASL Napoli 1 Centro, Napoli; 7 Centro Psiche Donna, Ambulatorio Psichiatrico P.O.M. Melloni, ASST Fatebenefratelli Sacco; 8 Aldo Ravelli Center for Nanotechnology and Neurostimulation, University of Milan, Milan, Italy; 9 Department of Psychiatry and Behavioral Sciences, Stanford University, Stanford, United States

## Abstract

**Introduction:**

Intranasal esketamine has emerged as an innovative treatment for TRD due to its rapid antidepressant effects. However, the clinical response is heterogeneous, and growing evidence suggests that comorbid personality disorders (PDs) may significantly shape treatment outcomes. Although PDs are frequently diagnosed in TRD patients, little is known about their differential influence on esketamine efficacy and response timelines.

**Objectives:**

This study aimed to explore distinct response trajectories across personality profiles and offer both neurobiological and psychodynamic interpretations.

**Methods:**

We conducted a prospective naturalistic observational study on 60 TRD patients treated with esketamine (56-84mg twice weekly for 4 weeks, then weekly thereafter). Patients were recruited from two specialized clinical centers in Northern Italy. All participants met DSM5 criteria for a PD assessed with the SCID-5-PD and were categorized into 4 subgroups: borderline (n = 15), obsessive-compulsive (n = 15), dependent (n = 15), and avoidant (n = 15). Depressive severity was assessed weekly using the MADRS from baseline (T0) to week 6.

**Results:**

Distinct patterns of clinical response emerged. Patients with:

- **dependent PD** showed rapid improvement from the 1st week, likely supported by the structured therapeutic setting and their externalized emotional regulation style.
- **borderline PD** demonstrated a pronounced early response (weeks 1–2) followed by relapse or affective instability around week 4–5, potentially reflecting idealization-devaluation dynamics towards treatment and the therapeutic team.
- **obsessive-compulsive PD** exhibited a slower but progressive improvement starting at week 3, possibly related to initial cognitive control and skepticism toward the treatment.
- **avoidant PD** had a moderate and steady response, likely influenced by ambivalence toward intimacy and fear of judgment within the therapeutic setting.

Statistical analysis revealed a significant time-group interaction (*p* < 0.001): the borderline group had the fastest early response and the obsessive-compulsive group showed the most stable improvements by week 3. Between-group effect size at week 4 was large (Cohen’s *d* = 1.52).

**Conclusions:**

These findings suggest that personality structure meaningfully influences esketamine treatment response – not only from a neurobiological perspective but also through relational and psychological processes. Dependent patients may benefit more rapidly due to the emotionally supportive context; obsessive-compulsive patients need time to relinquish cognitive control; and borderline patients, while initially reactive, may relapse due to unstable attachment dynamics. Integrating personality assessment and psychotherapeutic containment into esketamine protocols may improve efficacy and emotional safety, representing a key step towards genuinely personalized psychiatry.

**Disclosure of Interest:**

None Declared

